# Access to Telepharmacy Services May Reduce Hospital Admissions in Outpatient Populations During the COVID-19 Pandemic

**DOI:** 10.1089/tmj.2021.0420

**Published:** 2022-09-07

**Authors:** Erik Hefti, Benjamin Wei, Kristen Engelen

**Affiliations:** ^1^ Department of Pharmaceutical Sciences, Harrisburg University of Science and Technology, Harrisburg, Pennsylvania, USA.; ^2^ RxLive, Inc., St. Petersburg, Florida, USA.

**Keywords:** telepharmacy, hospital admission rates, outpatient, telehealth, outcomes, medication therapy management

## Abstract

**Introduction:**

Avoidable hospital admissions put increased pressure on already strained health care resources, causing emotional and financial distress for patients and their families while taxing the health system. Pharmacist involvement in patient care has been shown to improve health care outcomes. Telepharmacy allows for personalized interaction and access to pharmacy services in a flexible format. The primary aim of this report is to explore the impact that access to a personalized telepharmacy service has on the hospital admission rate in an outpatient population before and during the COVID-19 pandemic.

**Materials and Methods:**

A retrospective, double-arm cohort study was performed. Hospital admission rates were analyzed in two similarly aged groups; one group (*n* = 2,242) had access to telepharmacy services through their primary care provider and another group did not (*n* = 1,540), from 2019 to 2020. Statistical analysis was performed to explore hospitalization rates in both groups.

**Results:**

An increase in hospitalization rates was observed in both groups of patients from 2019 to 2020. The patient group that had access to the telepharmacy service demonstrated a reduced rise in hospitalization rates versus the group without access to the telepharmacy service (access group +12.9% vs. nonaccess group +40.2%,* p *< 0.05, Student's* t*-test).

**Discussion:**

The patient group with access to telepharmacy services demonstrated a reduced increase in hospitalizations versus the group without access in 2020. While this represents a preliminary investigation into the potential impacts of telepharmacy on hospitalization rates, telepharmacy services may have a role in improving patient outcomes and cost savings.

## Introduction

Preventable hospitalizations and adverse drug reactions (ADRs) are expensive events that can be taxing on the health care system and patients alike. The COVID-19 pandemic has further strained the health care system. The high cost of ADRs and associated hospitalizations has been documented beyond the United States and remains a global challenge.^1^

The cost of potentially preventable hospitalizations is measured in billions of dollars, with current estimates approaching $15 billion annually in the United States, which can be attributed to cardiovascular disease and diabetes mellitus alone, according to a 2016 study.^2^ Beyond uncontrolled disease states, ADRs also contribute to preventable hospitalizations, increased length of hospital stay, and overall mortality.^3^ A 2012 study estimated that ADRs attributed to injectable medications alone cost the U.S. taxpayer $2.7 to $5.1 billion annually for hospitalized patients.^4^

According to a 2019 meta-analysis, drug-related hospital admissions may constitute ∼15% of hospitalizations, many that were avoidable.^5^ A recent 2021 study indicated that 13% of preventable 30-day hospital readmissions were due to ADRs.^6^ Key ADRs that contribute to hospitalizations include inappropriate dosing, drug interactions, inaction on therapy modification based on abnormal laboratory values, lack of therapeutic drug monitoring, use of contraindicated drugs, and allergic reactions to medications.^7,8^ Among all ADR risk factors, those related to patient disease state and medication characteristics are among the most studied risk factors for experiencing an ADR.^9^

Pharmacists are members of the healthcare team who are responsible for the safe and effective use of medications. Pharmacists have been shown to reduce ADRs after hospitalization in outpatient patient populations.^10,11^ Pharmacists are trained to perform medication therapy management, medication monitoring, and advocacy functions that involve being a liaison between prescribers and patients. There are some mixed results when examining the primary literature regarding the impact pharmacy interventions have on patient outcomes. There are instances where pharmacist interventions do not demonstrate significant impacts on all measured outcomes in some patient populations.^12,13^

Pharmacist intervention and prescription review have been demonstrated to save health care dollars by optimizing medication regimens and preventing hospitalization in inpatient populations.^14^ Various patient populations may be benefited by pharmacist interventions and participation in care delivery, including patients with asthma, depression, type 2 diabetes mellitus, and hypertension.^12,15–18^ This makes pharmacists uniquely suited to address preventable hospitalizations due to ADRs.^19,20^

Pharmacists remain the most accessible health care professionals in the United States given the wide footprint of retail pharmacies.^21^ This accessibility can also impact the practical ability of pharmacists to personalize pharmacotherapeutic care for patients in need. Pharmacist accessibility can be further increased through approaches utilizing telepharmacy. Telepharmacy is the delivery of pharmaceutical care to patients using secured telecommunication through the telephone or internet and is the core component of telehealth. Telepharmacy allows for pharmacotherapy to be patient focused and can be delivered in a more comfortable environment for the patient. Access to personalized pharmacotherapy can be increased through telepharmacy services.^22^

Telepharmacy has been advocated by numerous health care and public health associations, including the American Society of Health-System Pharmacists.^23^ The COVID-19 pandemic has necessitated telepharmacy approaches to patient care. Telepharmacy is a safe and effective alternative to in-person pharmacotherapeutic care delivered by pharmacists.^24^ Preliminary studies have indicated that telepharmacy services improve safety in drug dispensing and reduce health care infrastructure burden.^25^

RxLive^®^ is a novel, active intervention telepharmacy service specifically developed to enhance the delivery of pharmaceutical care by pharmacists using modern telecommunication technology, specifically through telephone or televideo. RxLive pharmacists gather and utilize unique data captured through direct patient interaction. RxLive pharmacists can access and add to pertinent medical records, communicate with prescribers, generate and update medication lists, make recommendations to patients and their prescribers, and support multiple population health initiatives and prescribers' value-based program participation through this direct line of communication between the patient and the pharmacist.

Beyond serving as a platform to perform medication therapy management and reconciliation, the RxLive service also enables the pharmacist to document specific adherence challenges that a patient may report with a medication, including not only clinical issues but also the impact of pertinent social determinants of health. These impediments to medication adherence can include cost, access to care, burden of the regimen, health literacy, skills, potential and/or perceived side effects, and internal and external social and behavioral factors.

In this manner, RxLive pharmacists can discover and resolve subtle drug complications and interactions, flagging medications and issues for the prescriber to address as well as for a pharmacist to follow-up on later. The pharmacist can flag each medication as having an issue (but no change recommended), discontinuing the medication, changing the medication, or documenting other interventions. *Table 1* shows the possible interventions that pharmacists can make, which are also available to the provider following the consultation.

**Table 1.** Possible Interventions That Can Be Documented by the Pharmacist for Each Medication

ADHERENCE ISSUES			FINANCIAL ISSUES
SKILLS/UNDERSTANDING	SOCIAL FACTORS	COST/SIDE EFFECTS/ACCESS	
A challenge managing their multiple providers	A concern with the monitoring of their treatment	A challenge with access to care	Recommended a switch to a generic alternative
A health literacy issue	A lack of motivation	A challenge with continuity of care	Recommended a switch to a 90-day supply
A lack of knowledge about their illness	A relationship challenge with their provider	A challenge with the availability of health care professionals	Recommended a switch to a therapeutic alternative
A lack of knowledge about their therapy	A social/family support challenge	A problem with the intrusiveness of their medication regimen	Recommended a prescription for an OTC medication
A challenge in using their device	Limiting lifestyle factors	A problem with the pill burden of their medication regimen	Recommended an OTC medication
A communication skills challenge	Negative beliefs about the health care system	A problem with the specificity of their medication regimen	Found the same medication cheaper at an alternative pharmacy
A lack of confidence in their own capabilities	Psychological problems	A challenge with the cost of their medication regimen	Identified a copay assistance card
A problem with forgetfulness		A nonmanageable adverse event/side effect	Discontinued the medication
Physical difficulties		A potential prescribing error	Recommended a switch to an alternative dose
		Manageable adverse events/side effects	Recommended a switch to an alternative frequency
		A nonmanageable adverse event/side effect	Recommended a switch to a combination therapy

OTC, over-the-counter.

The interventions were adapted from the Health Level 7 working group clinical document architecture codes selected from Systematized Nomenclature of Medicine: Clinical Terms for pharmacist interventions.^26^

Financial difficulty is a common reason for nonadherence.^27,28^ The RxLive platform supports documentation of financial difficulties and other impediments to adherence, enabling the pharmacist to make recommendations for more effective pharmacotherapeutic regimens. This represents a patient-centered approach to optimize pharmacotherapy and bridge potential gaps that form between providers and patients.

Currently, there is a paucity of published information regarding the impact telepharmacy has on outpatient outcomes in general, including the impact on hospital admission rates. At the present time, there are no studies that investigate the impact of telepharmacy on hospitalization outcomes in an outpatient population. The COVID-19 pandemic adds urgency to investigating how telepharmacy can impact outcomes in outpatient populations.

The primary aim of this pilot study was to measure the impact that access to the novel telepharmacy platform, RxLive, has on hospital admission rates in a large outpatient population. Observations of this outcome took place before and during the COVID-19 pandemic. Elucidating the impact of telepharmacy services on hospitalization rates in outpatient populations may guide the efficient use of pharmacists as force multipliers in health care cost control and clinical outcome improvement.

## Materials and Methods

### STUDY DESIGN AND APPROACH

**Fig. 1.**
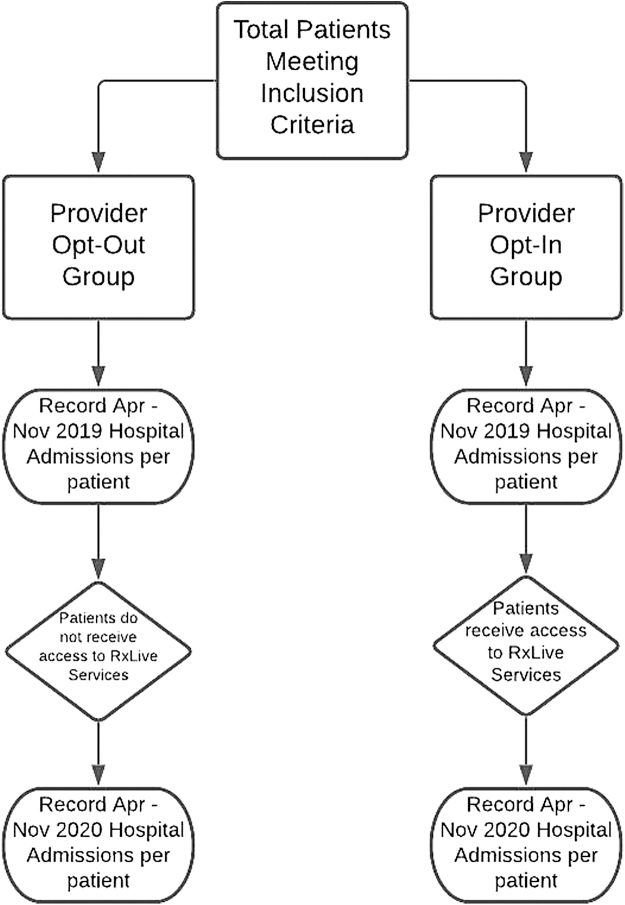
Study designed to analyze the impact of access to RxLive on the hospitalization rate in an outpatient population. The experimental architecture. The equivalent time period—a year before the RxLive provider enrollment—was 2019, which was compared with the 2020 period when the providers had access to RxLive enrollment.

This study utilized deidentified data for analysis and does not constitute human research. This research was IRB approved as exempt from human research requirements. A retrospective, double-arm cohort study was performed to investigate the potential impact that the ability to participate in the remote telepharmacy service, RxLive, had on hospitalization rates in an outpatient population. *Figure 1* summarizes the study architecture.

Patients of a primary care medical practice in Chester County, Pennsylvania, were selected for this study. The medical practice is preformed at six locations that provide primary care services to patients. A total of 28 primary care providers at this practice had the option of opting into the RxLive program or opting out. The 16 providers who opted into the RxLive program would prompt their patients to participate in the RxLive telepharmacy program based on inclusion criteria.

Inclusion criteria included patients who were prescribed six or more medications and had a primary care provider at the medical practice, and patients were eligible for this study throughout the study period. Patients who opted in would then be contacted and scheduled to meet with a pharmacist for comprehensive medication therapy management. The patients of the 12 nonparticipating providers were not prompted to participate in the RxLive program. These patients constituted the opt-out group for analysis purposes.

There were 2,242 patients in the opt-in group who had the ability to receive the RxLive service, starting at the “go-live” date of April 1, 2020, through the conclusion of the pilot period ending November 30, 2020. The hospitalization rate was analyzed and compared with the hospitalization rate observed during the same time period 1 year prior (April 1, 2019, to November 30, 2019) when the patients did not have the ability to utilize the RxLive service. There were 76 deaths recorded (3.4%) in the opt-in group.

There were 1,540 patients in the opt-out group who did not receive any RxLive services during the 2019 and 2020 observation periods and met the same inclusion criteria as the opt-in group. They were also assessed for hospitalizations in parallel with the opt-in group at the same interval. Changes in hospitalization rates in both groups could be compared for 2019 versus 2020 to measure the impact of RxLive services. There were 45 deaths recorded in the opt-out group (2.92%) in the observed time periods.

### STATISTICAL APPROACH

Data were analyzed using Microsoft Excel. Data are expressed as the mean ± standard deviation (SD). Two-tailed Student's *t*-tests were utilized to compare the hospitalization rates in both groups. Differences between means were considered to be significant at *p* < 0.05.

### PATIENT POPULATION AND DETERMINATION OF THE HOSPITALIZATION RATE

The mean age of patients in the opt-in group was 75.0 years (range = 22–103 years, SD = 9.52), and the mean age of the opt-out group was 74.0 years (range = 21–96 years, SD = 11.7). The mean age of each group was not only similar but also significantly different (*p* = 0.015, Student's *t*-test). The four most prevalent patient diagnoses in the opt-in and opt-out groups are shown in *Table 2*. A total of 830 patients in the opt-in group (37%) completed at least one consultation.

**Table 2.** Most Prevalent Comorbidities Present in the Opt-In and Opt-Out Groups

COHORT	TOTAL PATIENTS	CARDIOVASCULAR DISEASE [PERCENTAGE OF TOTAL]	DIABETES MELLITUS [PERCENTAGE OF TOTAL]	HYPERTENSIVE DISEASE [PERCENTAGE OF TOTAL]	MOOD DISORDERS [PERCENTAGE OF TOTAL]
Opt-in group	2,242	1,499 [66.9]	766 [34.2]	1,607 [71.7]	872 [38.9]
Opt-out group	1,540	604 [39.2]	406 [26.4]	702 [45.6]	367 [23.9]

A hospitalization is defined as any hospital admission in the observation period. Patients with multiple hospitalizations had each of their hospitalizations counted as an individual occurrence. The hospitalization rate was calculated as the total number of hospitalizations per patient in each respective group during the observation period.

## Results

A total of 1,083 consultations were performed in the opt-in group. From that total, 843 consultations were completed telephonically, while 183 consultations were completed using televideo. There were 57 consults where the communication method was not documented. A total of 14,657 medications were reviewed in the opt-in group and 1,773 interventions were documented. The hospitalization rates of the opt-in group versus the opt-out group during each assessment period are summarized in *Table 3*.

**Table 3.** Hospitalization Rates in the Opt-In and Opt-Out Groups During the 2019 and 2020 Assessment Periods

	TOTAL PATIENTS	HOSPITAL ADMISSIONS, NOVEMBER TO APRIL 2019	HOSPITALIZATIONS PER PATIENT, NOVEMBER TO APRIL 2019 [95% CI]	HOSPITAL ADMISSIONS, NOVEMBER TO APRIL 2020	HOSPITALIZATIONS PER PATIENT, NOVEMBER TO APRIL 2020 [95% CI]	CHANGE IN HOSPITALIZATION RATE FROM 2019 TO 2020
Opt-in group	2,242	482	0.21 [0.20–0.23]	544	0.24 [0.23–0.26]	+12.86%
Opt-out group	1,540	865	0.56 [0.54–0.59]	1213	0.79 [0.77–0.81]	+40.23%

**Fig. 2.**
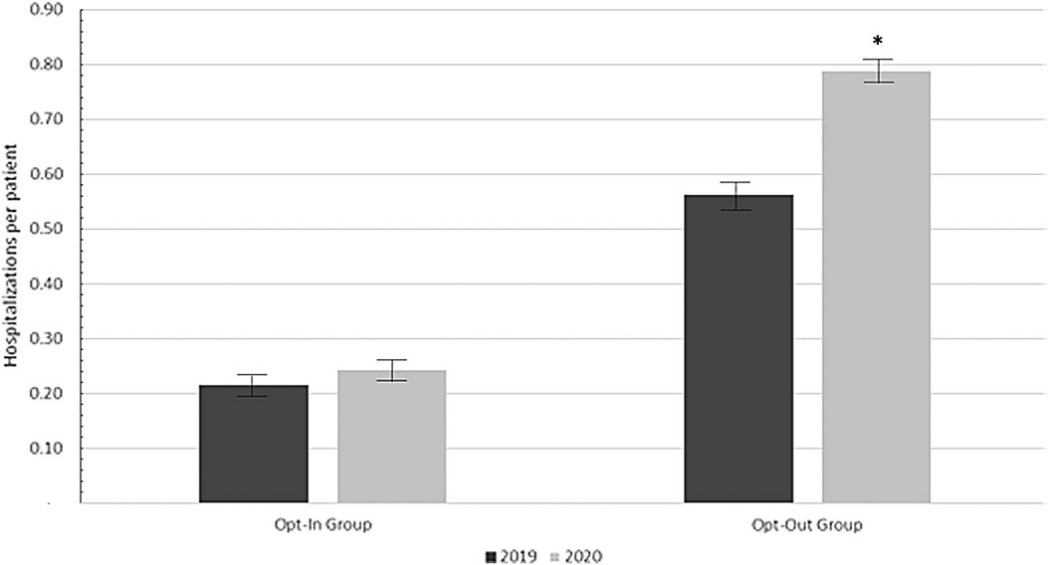
Time-adjusted hospital admissions per patient by provider cohort. The hospitalization rates for the opt-in and opt-out groups in the 2019 and 2020 observation periods. The error bars indicate 95% confidence intervals. There was a significantly higher rise in the hospitalization rate in the opt-out group from 2019 to 2020, as indicated by the asterisk (*).

The opt-out group exhibited a higher baseline hospitalization rate versus the opt-in group. Hospitalization rates were elevated in both the opt-in and opt-out groups in the 2020 assessment period versus the 2019 period. The opt-in group experienced a significantly lower elevation of hospitalizations from 2019 to 2020 compared with patients not enrolled in the program in the same periods (12.86% increase in the opt-in group vs. 40.23% increase in the opt-out group, Student's *t*-test, *p* = 0.001) (*Fig. 2*). This represents a (difference of 30.44%) lower rise in the hospitalization rate in the opt-in patient group with access to the RxLive service from 2019 to 2020.

## Discussion

Accessibility of RxLive services in outpatient populations was associated with a lower hospitalization rate, although both study groups displayed increased hospitalization rates in 2020 compared with 2019. These preliminary findings are consistent with other reports showing the benefit that telepharmacy can have on the overall well-being of various patient populations.^29,30^ Beyond the physical and emotional toll that hospitalizations have on patients, there is also a financial toll on the health care system and patient alike.

According to the Healthcare Cost and Utilization Project, the average cost of a hospitalization in the United States for adults over 65 years of age is ∼$12,000.^31,32^ The cost can vary based on the length of stay and nature of the admission diagnoses. The high cost of hospitalizations in the United States makes minimizing these events a critical marker of utility associated with a specific health care approach. In theory, if the hospitalization rate of the opt-out group in the 2020 assessment period was observed in the opt-in group, 675 total hospitalizations may have occurred.

The opt-in group only experienced a total of 544 hospitalizations in actuality. This results in a hypothetical avoidance of 131 hospitalizations in the opt-in group. Using the average of $12,000 per hospitalization, an estimated $1.57 million may have been avoided in the opt-in group. At the current stage, this conservative estimate not only represents a theoretical savings and needs to be further explored but also represents a future direction to quantify the value of this telepharmacy service.

While not specifically accounted for in the current study, there are numerous potential reasons the group of patients with access to the RxLive program experienced lower hospitalization rates. Pharmacist-provided pharmacotherapeutic care has been shown to reduce ADRs, improve patient education, and improve medication adherence.^33,34^ Delivering these aspects of pharmacotherapeutic care has been reported to reduce hospitalizations.^35^

It is reasonable to postulate that the RxLive program reduces hospitalization because it facilitates the delivery of pharmacotherapeutic care by pharmacists. The RxLive platform specifically prompts the pharmacist to address these key components of pharmacotherapeutic care for every patient. This reduces the likelihood of unaddressed medication problems that persist and could ultimately lead to hospitalization.

The COVID-19 pandemic has brought remote technology to the forefront of the health care discussion. The current study demonstrates a potential benefit of access to remote delivery of pharmaceutical care. Telehealth may allow for more efficient utilization of health care professionals' time and resources.^36,37^ Technology can act as a force multiplier for health care professionals to efficiently care for more patients who may not be able to travel for care.

The COVID-19 pandemic prevented many from accessing health care at a physical location. Telehealth approaches allowed clinicians to provide care in a safe accessible way to mitigate the limitations put in place during the pandemic.^38^ RxLive has the potential to positively impact patients' access to pharmaceutical care when access to physical locations is limited. Access to RxLive services during the COVID-19 pandemic also allowed for patients to self-isolate, decreasing potential direct interactions and exposure to the virus. It has been postulated by others that this aspect of telehealth has the potential to reduce morbidity and mortality in COVID-19 patients.^39^

During the pandemic, pharmacists received weekly continuing education on COVID-19 best practices. Furthermore, pharmacists disseminated up-to-date information regarding the status of the pandemic and ways to avoid infection to patients in the opt-in group. This information was based on recommendations from the Centers for Disease Control and Prevention (Supplementary File S1.) Whether this disseminated information regarding the COVID-19 pandemic played a role in the hospitalization trends observed is not currently known and further exploration into specific outcomes is a future aim.

It is possible that COVID-19 impacted hospitalization rates in both patient populations during the 2020 observation time frame. While COVID-19 may have been responsible for the overall rise in hospitalization rates seen in both groups, there are no indications that the opt-in and opt-out groups had any differences in risk factors that impacted acquiring the illness or its severity.

While telepharmacy platforms such as RxLive can increase patients' access to pharmaceutical care, there are limits that should be considered. RxLive is optimized for either telephonic or webcam-based patient interactions. This requires patients to have access to a telephone at a minimum. Generally, technology can act as a barrier to accessing telehealth services. A patient's socioeconomic status, age, geographic residence, mental health history, and technologic literacy can act as barriers to accessing telehealth services.^40^ This can also contribute to the overall health care disparities that telehealth seeks to alleviate.

The providers' practice methods and how state-of-the-art their approach to healthcare is may impact the patient outcomes. Currently, it is not possible to track adherence trends or provider acceptance of recommendations or interventions made by pharmacists, making longitudinal analysis of these variables absent from the current study. The impact of providers on hospitalization rates cannot be fully controlled for in the current study.

Patient involvement and participation in the delivery of health care have been shown to improve various clinical outcomes.^41,42^ As 37% of patients in the opt-in group chose to participate and receive a consultation, the opportunity to actively participate in health care processes may have contributed to the trends observed. The degree of involvement that patients in both groups have in their health care delivery was not assessed outside of utilization of RxLive services.

It should be noted that the current study is retrospective in approach and design. This makes prospective follow-up studies necessary to confirm any causal relationship between access to RxLive services and hospitalizations. In addition, being a retrospective study, there are potential clinical confounders in the patient population that may be impacting the hospitalization rate.

Comorbid disease states such as diabetes mellitus, congestive heart failure, renal failure, hypertension, and others may have impacted hospitalization rates and could not be controlled for in this pilot study. While comorbid conditions were recorded, they were not controlled for. Selection bias is also another potential confounder in the present study. Individuals who participate in telehealth consultations may be more engaged in their health care.

Accounting for age in the comparative groups does not replace accounting for other potential confounders that may impact hospitalization rates. Beyond comorbidities, patient engagement may be contributing to the results observed. Participation in telehealth consultations presents an additional opportunity for patient engagement. Patient engagement may also impact health care outcomes and may be contributing to the results seen in the present study.^42,43^

The opt-in group exhibited a lower hospitalization rate despite higher reported rates of cardiovascular disease, hypertensive disorders, diabetes mellitus, and mood disorders (*Table 2*). A future prospective approach that considers patients who were matched based on provider, sex, overall engagement, and clinical diagnoses could help reduce clinical confounders that may be present in the current study.

## Conclusions

This preliminary study showed the potential impact of efficient remote delivery of medication therapy management on hospitalization rates in an outpatient population during the COVID-19 pandemic. While additional prospective analysis is needed to corroborate these findings, telepharmacy services may benefit outpatient populations and health care infrastructure.

## Supplementary Material

Supplemental data

## References

[1] Chan SL, Ng HY, Sung C, et al. Economic burden of adverse drug reactions and potential for pharmacogenomic testing in Singaporean adults. Pharmacogenomics J 2019**;** 19:401–410. 30250149 10.1038/s41397-018-0053-1

[2] Sentell TL, Ahn HJ, Miyamura J, Juarez DT. Cost burden of potentially preventable hospitalizations for cardiovascular disease and diabetes for Asian Americans, Pacific Islanders, and Whites in Hawai'i. J Health Care Poor Underserved 2015**;** 26:63–82. 25981089 10.1353/hpu.2015.0068 PMC4554682

[3] Formica D, Sultana J, Cutroneo PM, et al. The economic burden of preventable adverse drug reactions: A systematic review of observational studies. Expert Opin Drug Saf 2018**;** 17:681–695. 29952667 10.1080/14740338.2018.1491547

[4] Lahue BJ, Pyenson B, Iwasaki K, et al. National burden of preventable adverse drug events associated with inpatient injectable medications: Healthcare and medical professional liability costs. Am Health Drug Benefits 2012**;** 5:1–10. 24991335 PMC4031698

[5] Ayalew MB, Tegegn HG, Abdela OA. Drug related hospital admissions; A systematic review of the recent literatures. Bull Emerg Trauma 2019**;** 7:339–346. 31857995 10.29252/beat-070401 PMC6911719

[6] Dalleur O, Beeler PE, Schnipper JL, Donzé J. 30-Day potentially avoidable readmissions due to adverse drug events. J Patient Saf 2021**;** 17:e379–e386. 28306610 10.1097/PTS.0000000000000346

[7] McDonnell PJ, Jacobs MR. Hospital admissions resulting from preventable adverse drug reactions. Ann Pharmacother 2002**;** 36:1331–1336. 12196047 10.1345/aph.1A333

[8] Preventable adverse drug reactions: A focus on drug interactions. 2020. Available at https://www.fda.gov/drugs/drug-interactions-labeling/preventable-adverse-drug-reactions-focus-drug-interactions (last accessed December 2, 2021).

[9] Zhou L, Rupa AP. Categorization and association analysis of risk factors for adverse drug events. Eur J Clin Pharmacol 2018**;** 74:389–404. 29222712 10.1007/s00228-017-2373-5

[10] Schnipper JL, Kirwin JL, Cotugno MC, et al. Role of pharmacist counseling in preventing adverse drug events after hospitalization. Arch Intern Med 2006**;** 166:565–571. 16534045 10.1001/archinte.166.5.565

[11] Murray MD, Ritchey ME, Wu J, Tu W. Effect of a pharmacist on adverse drug events and medication errors in outpatients with cardiovascular disease. Arch Intern Med 2009**;** 169:757–763. 19398687 10.1001/archinternmed.2009.59

[12] Adler DA, Bungay KM, Wilson IB, et al. The impact of a pharmacist intervention on 6-month outcomes in depressed primary care patients. Gen Hosp Psychiatry 2004**;** 26:199–209. 15121348 10.1016/j.genhosppsych.2003.08.005

[13] Machado M, Nassor N, Bajcar JM, et al. Sensitivity of patient outcomes to pharmacist interventions. Part III: Systematic review and meta-analysis in hyperlipidemia management. *Ann Pharmacother* 2008**;** 42:1195–1207. 18682540 10.1345/aph.1K618

[14] Jourdan JP, Muzard A, Goyer I, et al. Impact of pharmacist interventions on clinical outcome and cost avoidance in a university teaching hospital. Int J Clin Pharm 2018**;** 40:1474–1481. 30367375 10.1007/s11096-018-0733-6

[15] Lowrie R, Mair FS, Greenlaw N, et al. Pharmacist intervention in primary care to improve outcomes in patients with left ventricular systolic dysfunction. Eur Heart J 2012**;** 33:314–324. 22083873 10.1093/eurheartj/ehr433

[16] Machado M, Bajcar J, Guzzo GC, Einarson TR. Sensitivity of patient outcomes to pharmacist interventions. Part I: Systematic review and meta-analysis in diabetes management. *Ann Pharmacother* 2007**;** 41:1569–1582. 17712043 10.1345/aph.1K151

[17] Machado M, Bajcar J, Guzzo GC, Einarson TR. Hypertenion: Sensitivity of patient outcomes to pharmacist interventions. Part II: Systematic review and meta-analysis in hypertension management. *Ann Pharmacother* 2007**;** 41:1770–1781. 17925496 10.1345/aph.1K311

[18] Mehuys E, Van Bortel L, De Bolle L, et al. Effectiveness of pharmacist intervention for asthma control improvement. Eur Respir J 2008**;** 31:790–799. 18094011 10.1183/09031936.00112007

[19] Nicholls J, MacKenzie C, Braund R. Preventing drug-related adverse events following hospital discharge: The role of the pharmacist. Integr Pharm Res Pract 2017**;** 6:61–69. 29354552 10.2147/IPRP.S104639 PMC5774326

[20] Bushra R, Baloch SA, Jabeen A, et al. Adverse drug reactions: Factors and role of pharmacist in their prevention. J Ayub Med Coll Abbottabad 2015**;** 27:702–706. 26721044

[21] Manolakis PG, Skelton JB. Pharmacists' contributions to primary care in the United States collaborating to address unmet patient care needs: The emerging role for pharmacists to address the shortage of primary care providers. Am J Pharm Educ 2010**;** 74:S7. 21436916 10.5688/aj7410s7 PMC3058447

[22] Lam AY, Rose D. Telepharmacy services in an urban community health clinic system. J Am Pharm Assoc 2009**;** 49:652–659. 10.1331/JAPhA.2009.08128 19748874

[23] Alexander E, Butler CD, Darr A, et al. ASHP statement on telepharmacy. Am J Health Syst Pharm 2017**;** 74:e236–e241. 28438829 10.2146/ajhp170039

[24] Ameri A, Salmanizadeh F, Bahaadinbeigy K. Tele-pharmacy: A new opportunity for consultation during the COVID-19 pandemic. Health Policy Technol 2020**;** 9:281–282. 32837884 10.1016/j.hlpt.2020.06.005 PMC7293834

[25] Mohamed Ibrahim O, Ibrahim RM, Abdel-Qader DH, et al. Evaluation of telepharmacy services in light of COVID-19. Telemed J E Health 2021**;** 27:649–656. 33030986 10.1089/tmj.2020.0283

[26] Jindal N, Clifton C, Trahms K, et al. Community-based pharmacy use of the Pharmacist eCare Plan: A retrospective review. J Am Pharm Assoc (2003) 2021**;** 61:S161–S166. 33504447 10.1016/j.japh.2020.12.023

[27] Osborn CY, Kripalani S, Goggins KM, Wallston KA. Financial strain is associated with medication nonadherence and worse self-rated health among cardiovascular patients. J Health Care Poor Underserved 2017**;** 28:499–513. 28239015 10.1353/hpu.2017.0036 PMC5492520

[28] Moura LM, Schwamm EL, Moura Junior V, et al. Patient-reported financial barriers to adherence to treatment in neurology. Clinicoecon Outcomes Res 2016**;** 8:685–694. 27895506 10.2147/CEOR.S119971 PMC5117903

[29] Deas C, Stockton K. Evaluation of outcomes of a pharmacist-run, outpatient insulin titration telepharmacy service. Innov Pharm 2019**;** 10:24926/iip.v10.i2. 10.24926/iip.v10i2.1737 PMC7592856 34007540

[30] Vo AT, Gustafson DL. Telepharmacy in oncology care: A scoping review. J Telemed Telecare. 2020 [Epub ahead of print]; DOI: 10.1177/1357633X20975257 33377820

[31] Pfuntner A, Wier LM, Steiner C. Costs for hospital stays in the United States, 2010: statistical brief# 146. Healthcare cost and utilization project (HCUP) statistical briefs. Rockville, MD: Agency for Healthcare Research and Quality (US), 2006.

[32] Liang L, Moore B, Soni A. National Inpatient Hospital Costs: The Most Expensive Conditions by Payer, 2017: Statistical Brief# 261. Healthcare Cost and Utilization Project (HCUP) Statistical Briefs [Internet]. Rockville, MD: Agency for Healthcare Research and Quality, 2006. 32833416

[33] Bond C, Raehl CL. Clinical pharmacy services, pharmacy staffing, and adverse drug reactions in United States hospitals. Pharmacotherapy 2006**;** 26:735–747. 16716127 10.1592/phco.26.6.735

[34] Fisher RC. Patient education and compliance: A pharmacist's perspective. Patient Educ Couns 1992**;** 19:261–271. 1300624 10.1016/0738-3991(92)90145-9

[35] Col N, Fanale JE, Kronholm P. The role of medication noncompliance and adverse drug reactions in hospitalizations of the elderly. Arch Intern Med 1990**;** 150:841–845. 2327844

[36] Chakrabarti O. Telehealth: Emerging evidence on efficiency. Int Rev Econ Finance 2019**;** 60:257–264.

[37] Mercer L. Improving healthcare efficiency with telehealth. Available at https://www.healthrecoverysolutions.com/blog/improving-healthcare-efficiency-with-telehealth (last accessed December 7, 2021).

[38] Baum A, Kaboli PJ, Schwartz MD. Reduced in-person and increased telehealth outpatient visits during the COVID-19 pandemic. Ann Intern Med 2021**;** 174:129–131. 32776780 10.7326/M20-3026 PMC7429994

[39] Monaghesh E, Hajizadeh A. The role of telehealth during COVID-19 outbreak: A systematic review based on current evidence. BMC Public Health 2020**;** 20:1–9. 32738884 10.1186/s12889-020-09301-4 PMC7395209

[40] Lin C-CC, Dievler A, Robbins C, et al. Telehealth in health centers: Key adoption factors, barriers, and opportunities. Health Affairs 2018**;** 37:1967–1974. 30633683 10.1377/hlthaff.2018.05125

[41] Longtin Y, Sax H, Leape LL, et al. Patient participation: Current knowledge and applicability to patient safety. Mayo Clin Proc 2010**;** 85:53–62. 20042562 10.4065/mcp.2009.0248 PMC2800278

[42] Vahdat S, Hamzehgardeshi L, Hessam S, Hamzehgardeshi Z. Patient involvement in health care decision making: A review. Iran Red Crescent Med J 2014**;** 16:e12454. 24719703 10.5812/ircmj.12454 PMC3964421

[43] Bombard Y, Baker GR, Orlando E, et al. Engaging patients to improve quality of care: A systematic review. Implement Sci 2018**;** 13:98. 30045735 10.1186/s13012-018-0784-z PMC6060529

