# Supplementary material for: Access to Telepharmacy Services May Reduce Hospital Admissions in Outpatient Populations During the COVID-19 Pandemic
Source: Telemed J E Health. 2022 Sep 7;28(9):1324–31. doi: 10.1089/tmj.2021.0420 (PMC9508445; doi:10.1089/tmj.2021.0420)
Supplement: Supplemental data [file Supp_Data.docx]

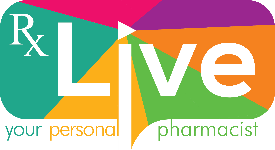


March 2020

**Pharmacist-led education and triage for COVID 19**

When speaking with patients for CMM, additional counseling should be provided at this time to mitigate patient concerns and call volumes into provider practices.

1. Strategies to manage current prescription medication supplies
   1. Request 90 day supplies
2. How to sign up for mail-order pharmacy may be appropriate for patients that cannot leave their homes. In the shared G drive you can find copies of mail order delivery sign-up forms that can be shared with patients.
3. How to manage risk factors
   1. Wash hands with soap for 20 seconds, followed by alcohol-based hand sanitizer
   2. Social distancing - stay home, keep 6 feet between yourself and others
4. Symptoms to look for in COVID 19 infection
   1. Cough, fever, difficulty breathing
5. Steps to take if a patient suspects exposure or infection by COVID 19
   1. Call nurse triage line at doctor’s office, and they will ask you a series of questions to help decide what next steps to take for assessment and treatment
   2. Flu and Strep are actively present in our communities and will likely need to be screened for if patients present with fever, cough, or respiratory symptoms, after first being screened by telephone

1. Supportive care and medication use to manage symptoms
   1. Stay hydrated
   2. Acetaminophen for fever. Recommendation to avoid NSAIDs developing- the reason: COVID binds to lung tissue enzyme ACE-II in the lungs and NSAIDs increase this enzyme, potentially facilitating the viral binding at lung tissue.
   3. Humidifier use
   4. Inhaler use as appropriate
